# Low baseline HbA1c and reduced eGFR are associated with relatively unfavorable body recomposition after SGLT2 inhibitor therapy in type 2 diabetes

**DOI:** 10.3389/fendo.2026.1924744

**Published:** 2026-08-27

**Authors:** Mizuki Gobaru, Nao Hasuzawa, Noboru Kurinami, Nobuhiko Wada, Lixiang Wang, Ayako Nagayama, Kenji Ashida, Yoshinori Moriyama, Hideaki Jinnouchi, Masatoshi Nomura

**Affiliations:** 1 Division of Endocrinology and Metabolism, Department of Internal Medicine, Kurume University School of Medicine, Kurume, Japan; 2 Diabetes Care Center, Jinnouchi Hospital, Kumamoto, Japan; 3 Department of Medical Biochemistry, Kurume University School of Medicine, Kurume, Japan

**Keywords:** body recomposition, fat mass, muscle mass, renal function, sarcopenia, SGLT2 inhibitors, type 2 diabetes mellitus

## Abstract

**Aims:**

Sodium-glucose cotransporter 2 inhibitors (SGLT2i) and glucagon-like peptide-1 receptor agonists (GLP-1RAs) improve glycemic control and promote weight loss, but their effects on the relative balance between skeletal muscle and fat mass remain incompletely understood. This study examined treatment-associated body composition changes in patients with type 2 diabetes mellitus (T2DM), focusing on muscle-to-fat balance.

**Methods:**

In this multicenter retrospective cohort study, we analyzed body composition changes 12 months after initiation of SGLT2i (n = 36), GLP-1RA (n = 17), or GLP-1RA add-on to SGLT2i therapy (n = 20). Changes in appendicular skeletal muscle mass (ΔASM) and body fat mass (ΔBFM) were assessed using bioelectrical impedance analysis. The Body Recomposition Score (BRS) was defined as ΔASM − ΔBFM and used as an exploratory index of the relative balance between muscle and fat mass changes; BRS < 0 was operationally defined as indicating relatively unfavorable body recomposition. An independent external cohort of patients with T2DM (n = 148) was used for external assessment.

**Results:**

ΔBFM and ΔASM were positively correlated with changes in body weight (ΔBW) in all treatment groups. However, ΔASM and ΔBFM were positively correlated only in the GLP-1RA group, whereas no such correlation was observed in the SGLT2i or GLP-1RA add-on to SGLT2i groups, suggesting interindividual heterogeneity in muscle-to-fat balance changes. In the SGLT2i group, BRS was positively correlated with baseline HbA1c (r = 0.36, p = 0.029) and estimated glomerular filtration rate (eGFR; r = 0.34, p = 0.040). No baseline variables correlated significantly with BRS in the other groups. Exploratory receiver operating characteristic analyses identified Youden index-derived cut-offs for discriminating BRS < 0 in the SGLT2i group: 6.6% for baseline HbA1c and 65 mL/min/1.73 m^2^ for eGFR. In the external cohort, BRS, calculated as the change in total skeletal muscle mass (ΔSMM) − ΔBFM at 4 weeks after SGLT2i initiation, showed significant positive correlations with baseline HbA1c and eGFR.

**Conclusions:**

Lower baseline HbA1c and reduced eGFR were associated with lower BRS after SGLT2i therapy in patients with T2DM. These exploratory findings support the importance of individualized pharmacotherapy for type 2 diabetes with consideration of muscle-to-fat balance.

**Clinical trial registration:**

## Graphical Abstract

**Figure:**
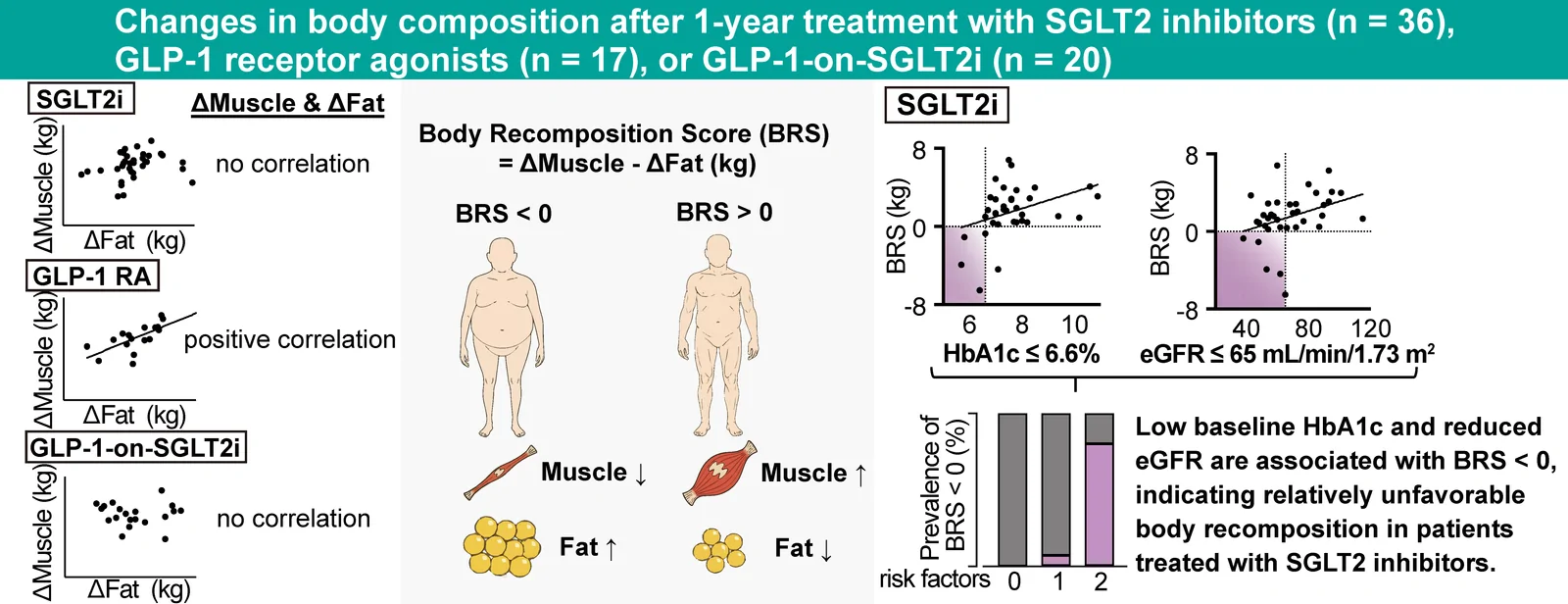
Body recomposition after 1 year of treatment with sodium-glucose cotransporter 2 inhibitors (SGLT2i), glucagon-like peptide-1 receptor agonists (GLP-1RAs), or GLP-1RA add-on to SGLT2i therapy (GLP-1-on-SGLT2i). Changes in appendicular skeletal muscle mass (ΔMuscle) and body fat mass (ΔFat) were positively correlated in the GLP-1RA group, but not in the SGLT2i or GLP-1-on-SGLT2i groups. Changes in muscle and fat mass were used to calculate the Body Recomposition Score (BRS = ΔMuscle − ΔFat, kg), an exploratory index of muscle-to-fat balance. Higher BRS values indicate greater preservation or gain of muscle mass relative to fat mass change. BRS < 0 was operationally defined as indicating relatively unfavorable body recomposition, in which muscle loss exceeded fat mass reduction or fat mass gain exceeded muscle gain. In patients treated with SGLT2i, lower baseline HbA1c and reduced eGFR were associated with lower BRS and a higher likelihood of BRS < 0.

## 1 Introduction

Type 2 diabetes mellitus (T2DM) is an increasingly prevalent global health concern, primarily driven by population aging and the rising incidence of obesity. The introduction of sodium-glucose cotransporter 2 inhibitors (SGLT2i) and glucagon-like peptide-1 receptor agonists (GLP-1RAs) has revolutionized the management of T2DM. Both drug classes have been shown to improve glycemic control, promote weight loss, and confer cardiovascular and renal protection (1–3).

SGLT2i lower blood glucose levels by inhibiting glucose reabsorption in renal proximal tubule cells, thereby promoting weight loss (4). Their cardiovascular and renal benefits have also been demonstrated in populations without diabetes, leading to their widespread use in clinical practice (5–8). GLP-1RAs improve glycemic control by enhancing glucose-dependent insulin secretion (9). They also delay gastric emptying, thereby attenuating postprandial glucose excursions, suppressing appetite, and promoting weight loss (10).

Several studies have evaluated the effects of SGLT2i and GLP-1RAs on body composition. Although the weight loss associated with both therapies predominantly reflects a reduction in fat mass in most studies, reductions in skeletal muscle mass have also been reported (11, 12). Some authors have suggested that changes in skeletal muscle mass may influence serum creatinine levels, which could affect the interpretation of renoprotective outcomes with SGLT2i (13).

The prevalence of sarcopenia is reported to be higher in T2DM patients than in healthy controls (14). Overnutrition and systemic inflammation promote protein catabolism and muscle atrophy, which in turn exacerbates insulin resistance (15). Emerging evidence highlights the clinical importance of body recomposition, commonly defined as the simultaneous reduction of body fat alongside the preservation or gain of lean mass, particularly in populations with metabolic diseases (16). Because adipose tissue and skeletal muscle play distinct but coordinated roles in systemic energy metabolism, pharmacological interventions for type 2 diabetes may alter the relative balance between fat and muscle mass. Given that sarcopenia impairs physical capacity to perform daily activities (17), the prevention and early management of sarcopenia are important for improving clinical outcomes and quality of life in patients with T2DM.

In this study, we therefore sought to examine treatment-associated changes in body composition after SGLT2i and GLP-1RA therapy by focusing on the balance between changes in muscle and fat mass.

## 2 Materials and methods

### 2.1 Study design and participants

This was a multicenter, retrospective cohort study based on the Diabetes Registry in Chikugo (DRC), which was registered in the UMIN Clinical Trial Registry under accession numbers UMIN000048469 (June 29, 2018) and UMIN000048471 (July 26, 2022). The source population comprised patients with diabetes mellitus who were treated at Kurume University Hospital and its affiliated institutions between June 2018 and April 2022. From this population, an internal cohort was defined based on the following inclusion criteria: (1) a diagnosis of T2DM; (2) age ≥ 20 years; (3) no prior use of SGLT2i or GLP-1RAs, with initiation of SGLT2i or GLP-1RAs during the observation period, or baseline use of an SGLT2 inhibitor with subsequent initiation of GLP-1RAs during the observation period; and (4) availability of body composition data obtained at the time of initiation and at 12 months after initiation of the target therapy. Patients who were hospitalized and underwent fasting management during the study period were excluded. SGLT2i included dapagliflozin, empagliflozin, and ipragliflozin, while GLP-1RAs included semaglutide (oral and injectable formulations), dulaglutide, and liraglutide.

In addition to the internal cohort, an independent external cohort derived from a separate study population was used for external assessment. This cohort comprised 148 patients with T2DM in whom changes in body composition were evaluated before and 4 weeks after the initiation of SGLT2i therapy, as described in our previously published study (18).

The internal cohort study was approved by the Ethics Committee of Kurume University School of Medicine (approval numbers 18053 and 22034). Written informed consent was waived; participants were provided with a written explanation of the study, and oral informed consent was obtained and documented in the medical records in accordance with the approved protocol. The external cohort consisted of a secondary analysis of previously collected clinical data. The present secondary analysis was approved by the Human Ethics Review Committee of Jinnouchi Hospital (approval number 2025-11-1), and the requirement for written informed consent was waived in accordance with the approved study protocol. All procedures were conducted in accordance with the Declaration of Helsinki.

### 2.2 Data collection

In the internal cohort, laboratory and body composition data were collected at the time of drug initiation and at 12 months. Laboratory parameters included hemoglobin A1c (HbA1c), blood urea nitrogen (BUN), serum creatinine (Cr), estimated glomerular filtration rate (eGFR), aspartate aminotransferase (AST), alanine aminotransferase (ALT), γ-glutamyl transpeptidase (γ-GTP), uric acid (UA), triglycerides (TG), low-density lipoprotein cholesterol (LDL-C), and high-density lipoprotein cholesterol (HDL-C). Laboratory analyses were performed at the hospital laboratory. Body composition parameters—including height, body weight, body mass index (BMI), appendicular skeletal muscle mass (ASM), body fat mass (BFM), and skeletal muscle index (SMI)—were assessed using a bioelectrical impedance analyzer (InBody 770; Biospace, Seoul, Korea).

In the external cohort, laboratory parameters and body composition data (body weight, BMI, total skeletal muscle mass [SMM], and BFM) were collected at the time of drug initiation and at 4 weeks thereafter using a bioelectrical impedance analyzer (InBody 770; Biospace, Seoul, Korea).

### 2.3 Statistical analysis

Data were presented as means ± standard deviations (SD) for continuous variables and as numbers and percentages for categorical variables, unless otherwise indicated. Within-group comparisons before and after drug initiation were performed using the Wilcoxon signed-rank test because normality was not met for several parameters. For comparisons among the three groups in the internal cohort at baseline and 12 months after drug initiation, one-way analysis of variance (ANOVA) was used for continuous variables, and the chi-square test or Fisher’s exact test was used for categorical variables, as appropriate. For comparisons of BRS in **Supplementary Figure 1**, the Kruskal–Wallis test or unpaired t-test was used, depending on the comparison and the results of normality analysis. To compare parameters between patients stratified within each treatment group by baseline HbA1c or baseline eGFR, the Mann–Whitney U test was used. Pearson’s correlation coefficients were calculated to assess associations between two continuous variables. Spearman’s rank correlation analyses were also performed as sensitivity analyses to assess the robustness of the Pearson correlation analyses. Receiver operating characteristic (ROC) analyses were performed to evaluate the discriminatory ability of baseline variables for identifying patients with BRS < 0, and cut-off values were determined using the Youden index. All statistical tests were two-sided, and p values < 0.05 were considered statistically significant. All analyses were performed using JMP Pro^®^ version 18.2.2 (SAS Institute Inc., Cary, NC, USA) and GraphPad Prism version 10.

## 3 Results

### 3.1 Patient characteristics

From the source population, a total of 73 patients who met the inclusion criteria were identified. This internal cohort comprised 36 patients in the SGLT2i group, 17 in the GLP-1RA group, and 20 in the GLP-1RA add-on to SGLT2i (GLP-1-on-SGLT2i) group. Baseline characteristics are summarized in **Table 1**. No statistically significant differences were observed between the groups in terms of age, sex, or the baseline use of oral glucose-lowering agents.

**Table 1:** Baseline characteristics of the internal cohort (N = 73).

	SGLT2i (n = 36)	GLP-1RA (n = 17)	GLP-1-on-SGLT2i (n = 20)	*p*
Age (years)	61.8 ± 14.3 (65 [51.5–72.8])	63.5 ± 8.8 (66 [58–70])	57.1 ± 13.4 (57.5 [51.5–67])	0.28
Sex, n (%) (male)	19 (52.8)	7 (41.2)	12 (60.0)	0.52
Diabetes medications in use, n (%)				
Biguanide	22 (61.1)	11 (64.7)	8 (40.0)	0.24
DPP-4 inhibitor	16 (44.4)	6 (35.3)	6 (30.0)	0.54
Sulfonylurea	9 (25.0)	3 (17.7)	5 (25.0)	0.88
Glinide	0 (0)	1 (5.9)	0 (0)	0.23
α-glucosidase inhibitor	0 (0)	0 (0)	2 (10.0)	0.12
Insulin	5 (13.9)	6 (35.3)	6 (30.0)	0.16

Data are presented as mean ± standard deviation and median [interquartile range] for continuous variables, or number (%). Values in parentheses below the mean ± standard deviation indicate median [interquartile range]. Baseline comparisons of age were performed using one-way analysis of variance (ANOVA). Comparisons of sex and medication use among groups were conducted using the chi-square test and Fisher’s exact test, as appropriate.

SGLT2i, sodium–glucose cotransporter 2 inhibitor; GLP-1RA, glucagon-like peptide-1 receptor agonist.

### 3.2 Clinical parameters before and after 12 months of therapy

Clinical and laboratory parameters at baseline and at 12 months after initiation of therapy are presented in **Supplementary Table 1**. Between-group comparisons of baseline and 12-month values were conducted to characterize group-specific profiles prior to analyses of changes from baseline. HbA1c and serum uric acid levels differed significantly among the groups at baseline. HbA1c was 7.6 ± 1.2% [59 ± 13 mmol/mol] in the SGLT2i group, 9.0 ± 2.0% [75 ± 22 mmol/mol] in the GLP-1RA group, and 8.1 ± 1.0% [65 ± 11 mmol/mol] in the GLP-1-on-SGLT2i group. Serum uric acid levels were 8.7 ± 5.9 mg/dL, 5.5 ± 1.3 mg/dL, and 5.4 ± 1.8 mg/dL, respectively. No other baseline parameters showed significant between-group differences.

Following 12 months of therapy, significant reductions in HbA1c were observed in all three groups, and the between-group differences in HbA1c and serum uric acid observed at baseline persisted at 12 months. Within-group analyses showed significant decreases in AST, ALT, and γ-GTP in both the SGLT2i group and the GLP-1-on-SGLT2i group; a significant decrease in TG was observed only in the SGLT2i group. A significant decrease in body weight and in body fat mass was observed in all three groups.

### 3.3 Treatment-associated changes in fat and muscle, and their associations with baseline characteristics

**Figure 1:**
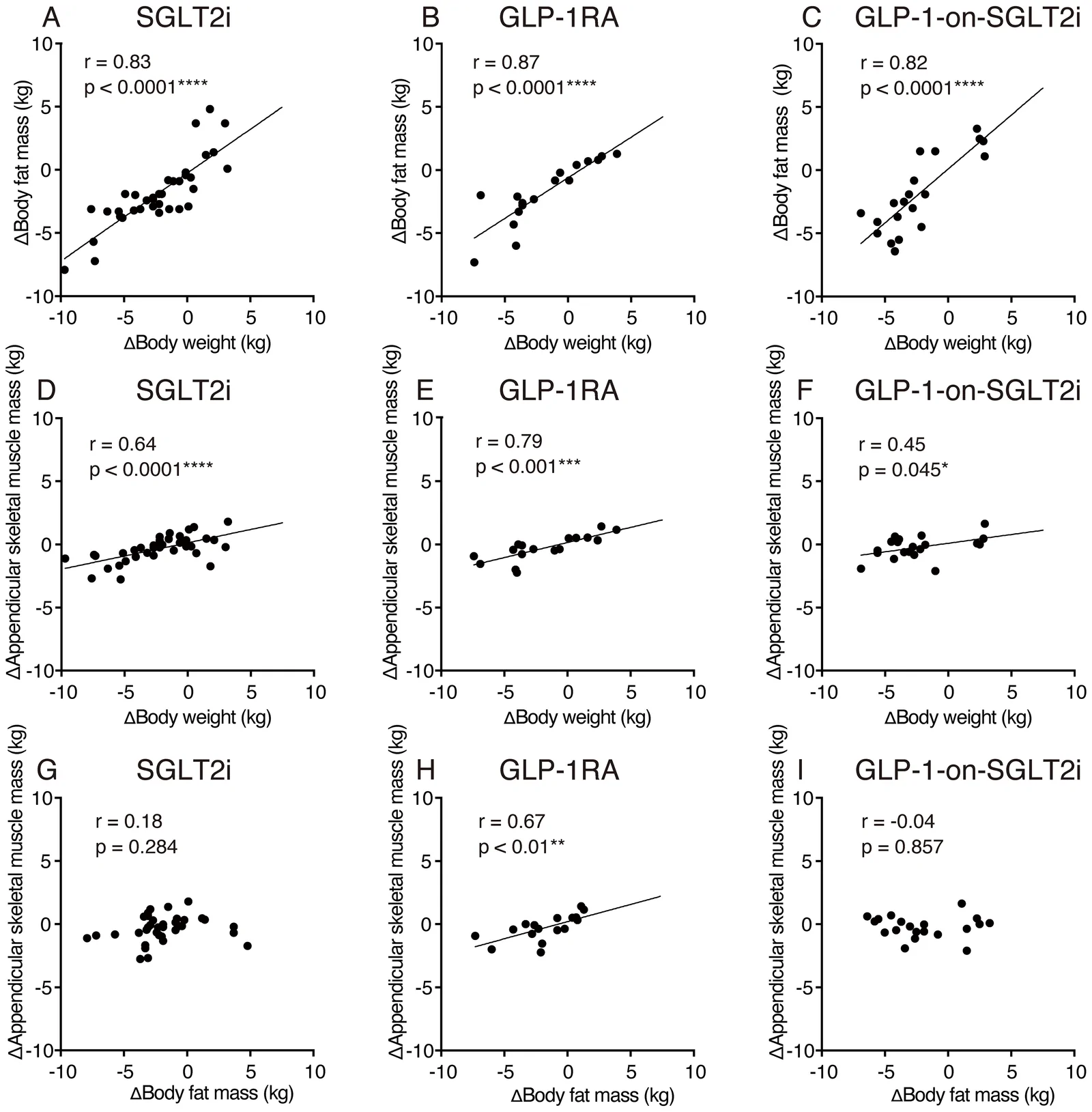
Correlations between changes in body weight, body fat mass, and appendicular skeletal muscle mass. **(A–I)** Scatter plots show the correlations between changes in body weight (ΔBW), body fat mass (ΔBFM), and appendicular skeletal muscle mass (ΔASM) over 12 months in the SGLT2i (n = 36), GLP-1RA (n = 17), and GLP-1-on-SGLT2i groups (n = 20). Pearson’s correlation coefficients (r) and corresponding p values are shown in each panel. Solid lines represent linear regression fits for statistically significant correlations. BW, body weight; ASM, appendicular skeletal muscle mass; BFM, body fat mass; SGLT2i, sodium–glucose cotransporter 2 inhibitor; GLP-1RA, glucagon-like peptide-1 receptor agonist.

To characterize treatment-associated changes in body composition, we analyzed correlations between changes in body weight (ΔBW), appendicular skeletal muscle mass (ΔASM), and body fat mass (ΔBFM) in each group (**Figure 1**). In all three groups, both ΔBFM and ΔASM showed significant positive correlations with ΔBW (**Figures 1A–F**). However, a significant correlation between ΔASM and ΔBFM was observed only in the GLP-1RA group, while no such correlation was found in the SGLT2i or GLP-1-on-SGLT2i groups (**Figures 1G–I**). These findings suggest that GLP-1RA therapy is associated with proportionate changes in muscle and fat mass, whereas SGLT2i-based regimens show inter-individual variability in the relationship between muscle and fat mass changes.

To address this variability, we next examined associations between patients’ baseline characteristics and body composition changes (ΔASM and ΔBFM) within each group. **Supplementary Table 2** summarizes the Pearson correlation coefficients and corresponding p values between baseline variables and ΔASM or ΔBFM. In the SGLT2i group, ΔBFM showed a significant negative correlation with baseline HbA1c, and a trend toward a negative correlation with eGFR, suggesting that negative ΔBFM values (i.e., greater fat loss) may be associated with higher baseline HbA1c and eGFR levels. In contrast, no significant correlations were found between baseline variables and ΔASM or ΔBFM in the GLP-1RA or GLP-1-on-SGLT2i groups.

### 3.4 Body recomposition assessed by the body recomposition score and its associations with baseline characteristics

Given that body recomposition—defined as a relative reduction in fat mass with preservation or gain of skeletal muscle—is generally considered beneficial for the management of T2DM (19), we defined the Body Recomposition Score (BRS = ΔASM − ΔBFM) as an exploratory and operational index of the relative balance between changes in skeletal muscle and fat mass. Higher BRS values indicate greater preservation or gain of ASM relative to changes in BFM. BRS < 0 was operationally defined as a relatively unfavorable muscle-to-fat balance, representing a pattern in which skeletal muscle loss exceeded fat mass reduction or fat mass gain exceeded skeletal muscle gain.

Among the 73 patients in the internal cohort, the mean BRS was 1.58 ± 2.64 kg (SGLT2i: 1.53 ± 2.65 kg; GLP-1RA: 1.50 ± 1.96 kg; GLP-1-on-SGLT2i: 1.73 ± 3.19 kg), with no significant difference among groups, suggesting that, on average, all treatment groups showed positive relative muscle-to-fat balance changes as assessed by BRS (**Supplementary Figure 1**). No significant differences in BRS were observed between male and female patients. However, 15 patients (20.5% of the internal cohort; five in each group) exhibited BRS < 0 kg, indicating a relatively unfavorable muscle-to-fat balance. Among them, five patients (6.8% of the internal cohort; three in the SGLT2i group and two in the GLP-1-on-SGLT2i group) had BRS < −3 kg, suggesting a more marked lower-tail imbalance in body recomposition.

**Figure 2:**
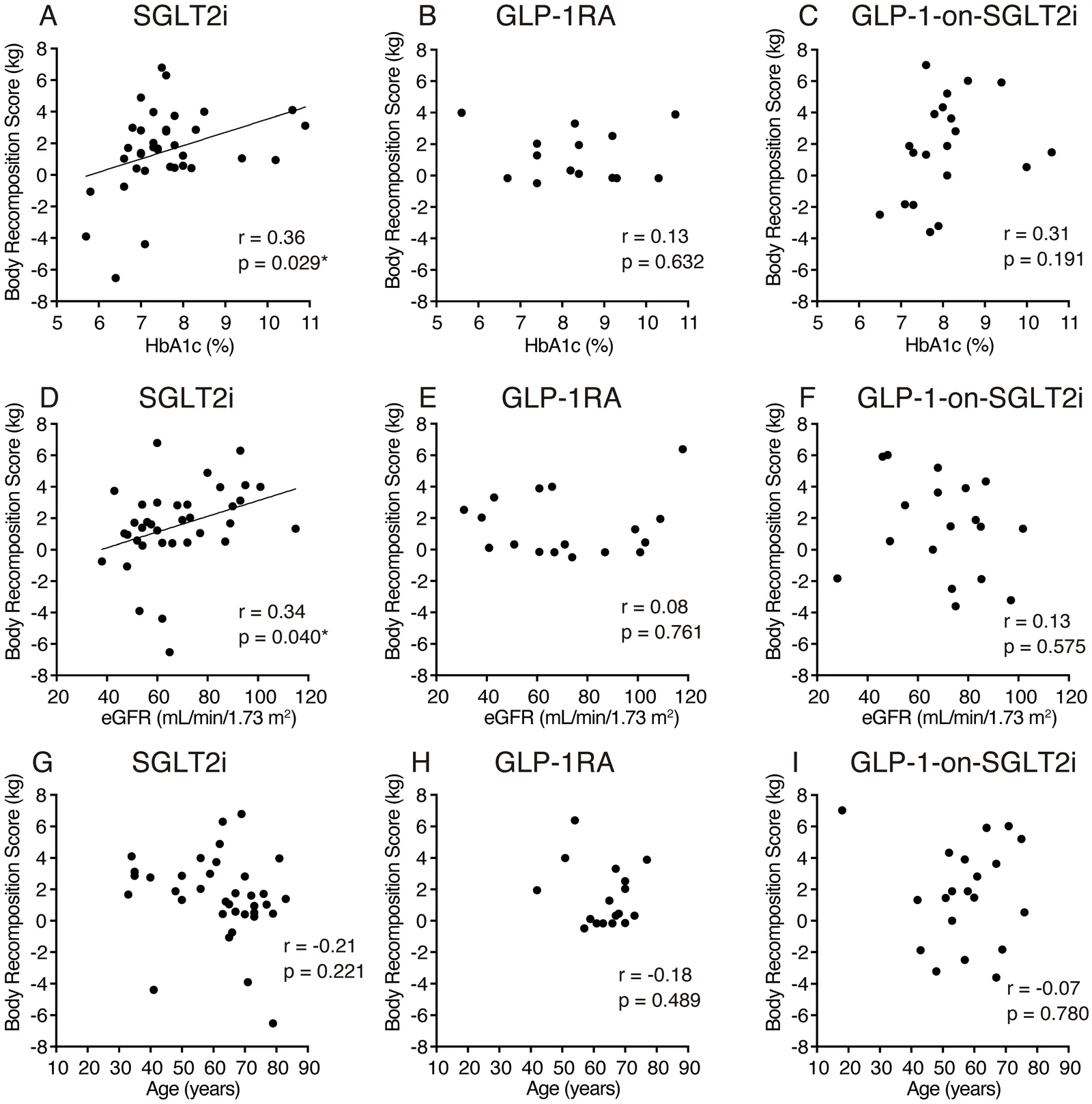
Correlations between baseline clinical variables and Body Recomposition Score. Scatter plots show the correlations of baseline HbA1c **(A–C)**, eGFR **(D–F)**, and age **(G–I)** with the Body Recomposition Score (BRS) over 12 months in the SGLT2i (n = 36), GLP-1RA (n = 17), and GLP-1-on-SGLT2i (n = 20) groups. Pearson’s correlation coefficients (r) and corresponding p values are shown in each panel. Solid lines represent linear regression fits for statistically significant correlations. Spearman’s rank correlation analyses performed as sensitivity analyses are shown in **Supplementary Table 4**. BRS, Body Recomposition Score (ΔASM − ΔBFM); ASM, appendicular skeletal muscle mass; BFM, body fat mass; eGFR, estimated glomerular filtration rate; SGLT2i, sodium–glucose cotransporter 2 inhibitor; GLP-1RA, glucagon-like peptide-1 receptor agonist.

To explore baseline characteristics associated with low BRS values, we performed univariate correlation analyses between BRS and baseline variables (HbA1c, eGFR, body weight, age, skeletal muscle mass, and body fat mass) within each group. The results are shown in **Figure 2** and **Supplementary Table 3**. In the SGLT2i group, both baseline HbA1c (r = 0.36, p = 0.029) and eGFR (r = 0.34, p = 0.040) were positively correlated with BRS, indicating that lower baseline HbA1c and eGFR values were associated with lower BRS, suggestive of a relatively unfavorable muscle-to-fat balance (**Figures 2A, D**). Spearman’s rank correlation analyses performed as sensitivity analyses showed similar positive correlations of BRS with baseline HbA1c (ρ = 0.37, p = 0.027) and eGFR (ρ = 0.42, p = 0.011) in the SGLT2i group (**Supplementary Table 4**). Age showed no significant association with BRS in Pearson correlation analyses, but showed a significant negative correlation in Spearman’s rank correlation analysis in the SGLT2i group (ρ = −0.39, p = 0.018; **Supplementary Table 4**). In contrast, none of the baseline variables showed significant correlations with BRS in the GLP-1RA or GLP-1-on-SGLT2i groups (**Figures 2B, C, E, F, H, I**; **Supplementary Tables 3**, **4**).

### 3.5 Discriminatory ability of baseline HbA1c and eGFR for BRS < 0

**Figure 3:**
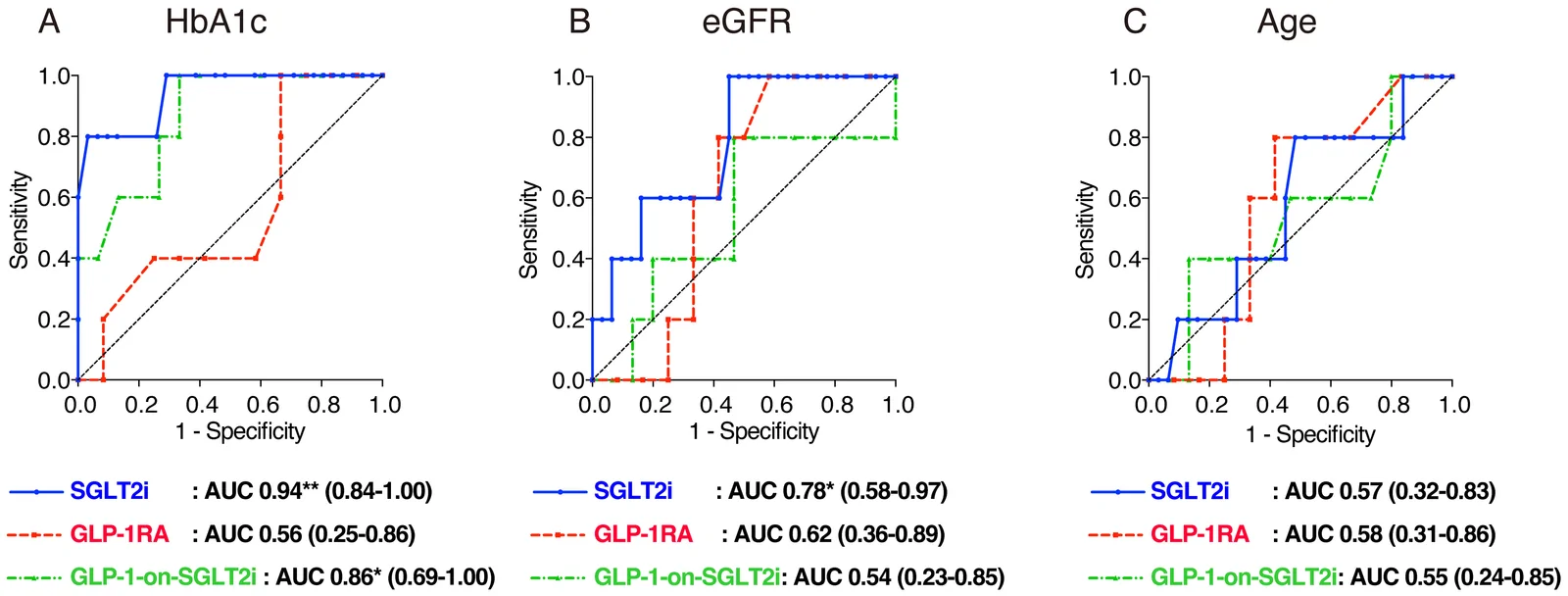
ROC curves of baseline HbA1c, eGFR, and age for discriminating BRS < 0. Receiver operating characteristic (ROC) analyses were performed to evaluate the ability of baseline HbA1c **(A)**, eGFR **(B)**, and age **(C)** to discriminate Body Recomposition Score (BRS = ΔASM − ΔBFM) < 0, in the SGLT2i (n = 36), GLP-1RA (n = 17), and GLP-1-on-SGLT2i (n = 20) groups. AUC values are shown with 95% confidence intervals in the figure. *p < 0.05, **p < 0.01 vs. AUC = 0.5. ASM, appendicular skeletal muscle mass; BFM, body fat mass; SGLT2i, sodium-glucose cotransporter 2 inhibitor; GLP-1RA, glucagon-like peptide-1 receptor agonist.

As BRS < 0 was operationally defined as indicating a relatively unfavorable muscle-to-fat balance, we performed receiver operating characteristic (ROC) analyses to evaluate the ability of baseline HbA1c, eGFR, and age to discriminate patients exhibiting BRS < 0 (**Figures 3A–C**; **Supplementary Table 5**). In the SGLT2i and GLP-1-on-SGLT2i groups, HbA1c showed relatively high AUCs of 0.94 and 0.86, respectively. eGFR showed moderate discriminatory ability only in the SGLT2i group (AUC = 0.78). Age did not show discriminatory utility in any group. The exploratory cut-off values for discriminating BRS < 0 according to this ROC analysis are summarized in **Supplementary Table 5**. In the SGLT2i group, the Youden index-derived cut-off values were 6.6% [49 mmol/mol] for HbA1c and 65 mL/min/1.73 m^2^ for eGFR.

**Figure 4:**
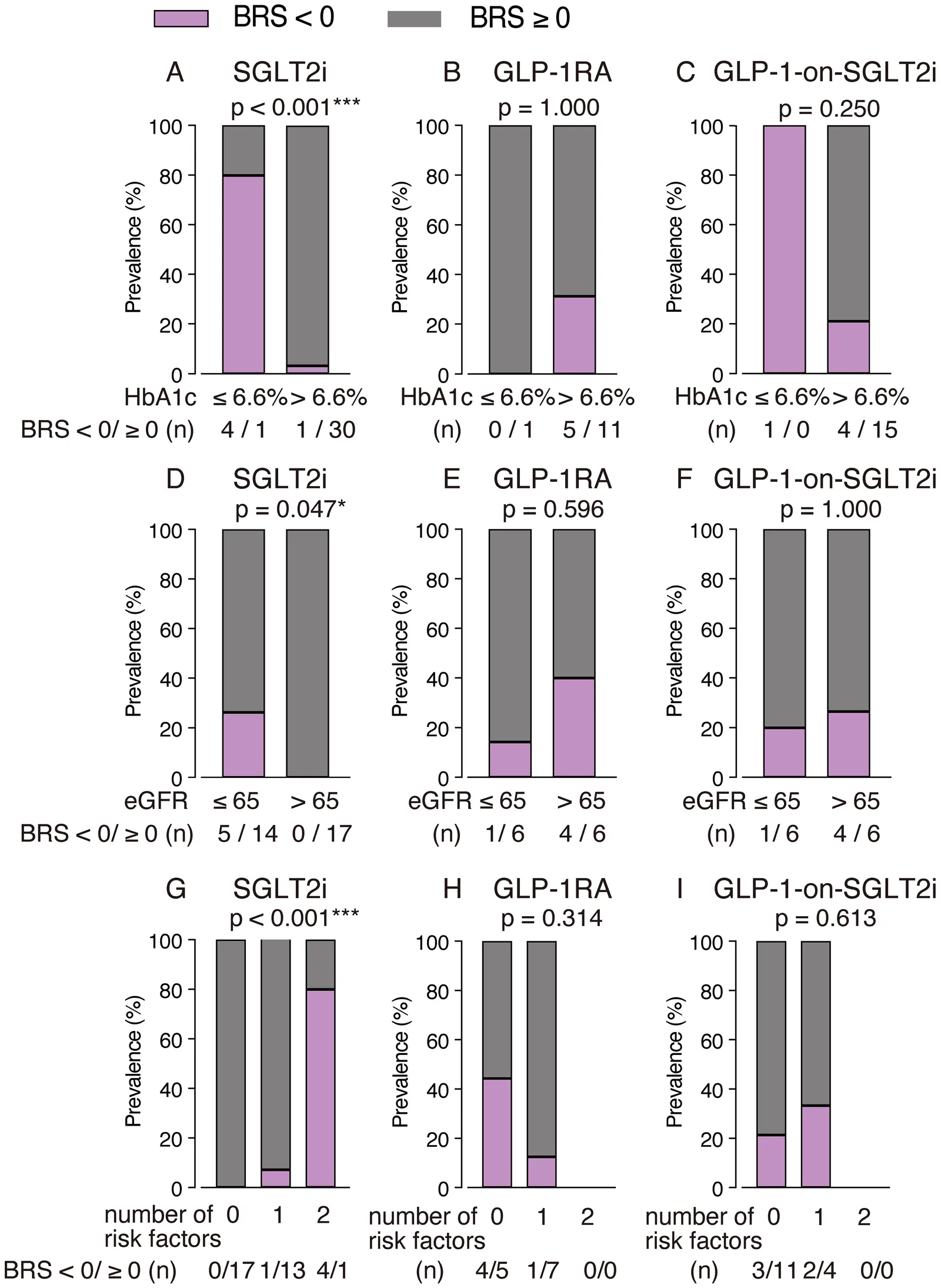
Associations of baseline HbA1c and eGFR with the prevalence of BRS < 0. The prevalence of BRS (= ΔASM − ΔBFM) < 0 was compared between patients stratified by baseline HbA1c (≤ 6.6% vs > 6.6%) and, separately, eGFR (≤ 65 vs > 65 mL/min/1.73 m^2^) in the SGLT2i, GLP-1RA, and GLP-1-on-SGLT2i groups **(A–F)**. The lower panels show the prevalence of BRS < 0 according to the number of candidate risk factors, defined as HbA1c ≤ 6.6% and eGFR ≤ 65 mL/min/1.73 m^2^ (0, 1, or 2 risk factors) **(G–I)**. Numbers below the x-axis indicate the number of patients with BRS < 0 and BRS ≥ 0 in each subgroup. Group comparisons were performed using Fisher’s exact test. BRS, Body Recomposition Score; ASM, appendicular skeletal muscle mass; BFM, body fat mass; eGFR, estimated glomerular filtration rate; SGLT2i, sodium–glucose cotransporter 2 inhibitor; GLP-1RA, glucagon-like peptide-1 receptor agonist. *p < 0.05; ***p < 0.001.

When patients were dichotomized based on these respective cut-off values identified in the SGLT2i group, both parameters were significantly associated with the prevalence of BRS < 0 in the SGLT2i group, but not in the GLP-1RA or GLP-1-on-SGLT2i groups (**Figures 4A–F**). BRS and other body composition–related parameters were compared between patients stratified by baseline HbA1c (≤ 6.6% vs > 6.6%) or by baseline eGFR (≤ 65 vs > 65 mL/min/1.73 m^2^) (**Supplementary Table 6**). In the SGLT2i group, BRS and ΔBFM differed significantly when patients were stratified by either baseline HbA1c or baseline eGFR. With regard to BRS and its components, no significant differences were observed in the GLP-1RA or GLP-1-on-SGLT2i groups after stratification by baseline HbA1c or eGFR, except that ΔASM differed significantly between eGFR strata in the GLP-1-on-SGLT2i group, with a positive ΔASM observed in the eGFR ≤ 65 mL/min/1.73 m^2^ subgroup. When baseline HbA1c ≤ 6.6% and eGFR ≤ 65 mL/min/1.73 m^2^ were treated as candidate risk factors, the prevalence of BRS < 0 in the SGLT2i group was 0% (0/17), 7.1% (1/14), and 80% (4/5) in patients with neither, one, or both candidate risk factors, respectively (**Figure 4G**, p < 0.001). In contrast, no significant differences in the prevalence of BRS < 0 were observed between patients with no candidate risk factor and those with one candidate risk factor in either the GLP-1RA or GLP-1-on-SGLT2i group (**Figures 4H, I**).

### 3.6 External cohort assessment

For external assessment of the associations between BRS and eGFR or HbA1c observed in the SGLT2i group of the internal cohort, we used data from an independent external cohort reported in our previous study (18). This cohort consisted of 148 patients with type 2 diabetes mellitus, in whom body composition changes were assessed at baseline and at 4 weeks after initiation of SGLT2i therapy, reflecting early changes after treatment initiation. In this external cohort, BRS was calculated using total skeletal muscle mass (SMM) as BRS = ΔSMM − ΔBFM due to the unavailability of appendicular skeletal muscle mass.

**Figure 5:**
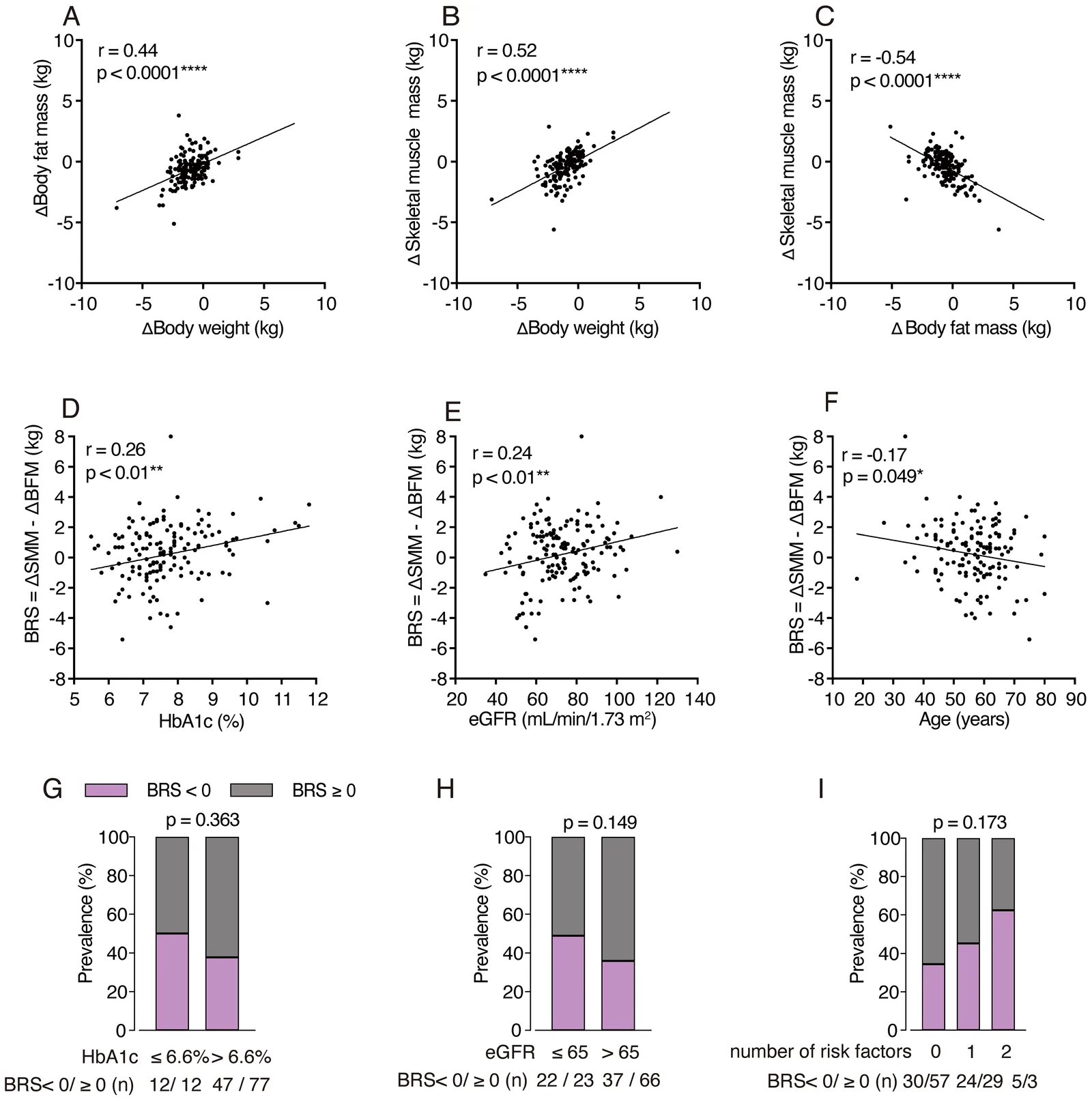
External cohort assessment of body recomposition patterns after SGLT2 inhibitor initiation. Scatter plots show the correlations among changes in body weight (ΔBW), body fat mass (ΔBFM), and total skeletal muscle mass (ΔSMM) at 4 weeks after initiation of SGLT2i therapy in the external cohort **(A–C)**. Scatter plots also show the associations between Body Recomposition Score (BRS = ΔSMM − ΔBFM) and baseline HbA1c, eGFR, and age **(D–F)**. Pearson’s correlation coefficients (r) and corresponding p values are shown in each panel. Solid lines indicate linear regression fits for statistically significant correlations. The lower panels show the prevalence of BRS < 0 stratified by baseline HbA1c (≤ 6.6% vs > 6.6%), baseline eGFR (≤ 65 vs > 65 mL/min/1.73 m^2^), and the number of candidate risk factors (HbA1c ≤ 6.6% and eGFR ≤ 65 mL/min/1.73 m^2^; 0, 1, or 2) **(G–I)**. Numbers below the x-axis indicate the number of patients with BRS < 0 and BRS ≥ 0 in each subgroup. Group comparisons were performed using Fisher’s exact test. BRS, Body Recomposition Score; BW, body weight; SMM, total skeletal muscle mass; BFM, body fat mass; eGFR, estimated glomerular filtration rate; HbA1c, hemoglobin A1c; SGLT2i, sodium–glucose cotransporter 2 inhibitor. *p < 0.05; **p < 0.01; ****p < 0.0001.

Clinical and body composition parameters at baseline and at 4 weeks are presented in **Table 2**. As shown in **Figures 5A, B**, both ΔBFM and ΔSMM at 4 weeks after SGLT2i initiation showed significant positive correlations with ΔBW. In addition, ΔSMM and ΔBFM showed a significant negative correlation, suggesting disproportionate changes in muscle and fat balance following SGLT2i treatment (**Figure 5C**). BRS showed statistically significant positive correlations with both HbA1c and eGFR, showing directional consistency with the continuous association analyses in the internal cohort (**Figures 5D, E**). Moreover, a significant negative correlation was also observed between BRS and age, suggesting that older age may be associated with a lower BRS in this cohort (**Figure 5F**).

**Table 2:** Clinical and body composition parameters at baseline and 4 weeks after initiation of SGLT2 inhibitor therapy in the external cohort (n = 148).

	Baseline	4W	*p*
Age (years)	56.4 ± 10.4 (58 [50–63])	—	
Sex, n (%) (male)	107 (72.3)	—	
eGFR (mL/min/1.73 m^2^)	73.0 ± 16.0 (71.4 [62.2–83.9])	—	
HbA1c (%)/(mmol/mol)	7.7 ± 1.2/61 ± 13 (7.5 [6.9–8.3]/58 [52–67])	7.3 ± 1.0/56 ± 12 (7.2 [6.7–7.8]/55 [50–62])	< 0.0001****
body weight (kg)	77.1 ± 15.3 (76.4 [65.9–85.6])	76.1 ± 15.2 (75.1 [65.3–84.2])	< 0.0001****
BMI (kg/m^2^)	27.9 ± 4.5 (27.5 [25.0–30.3])	27.5 ± 4.5 (27.0 [24.6–29.7])	< 0.0001****
Total skeletal muscle mass (kg)	48.9 ± 8.7 (49.0 [42.6–54.5])	48.5 ± 8.8 (48.8 [42.0–54.2])	0.0002***
Body fat mass (kg)	25.4 ± 9.6 (25.0 [18.0–30.6])	24.8 ± 9.6 (23.9 [17.6–30.0])	<0.0001****

Data are presented as mean ± standard deviation and median [interquartile range] for continuous variables, or number (percentage), as appropriate. Values in parentheses below the mean ± standard deviation indicate median [interquartile range]. Paired comparisons between baseline and 4 weeks after initiation of SGLT2 inhibitor therapy were conducted using the Wilcoxon signed-rank test. *p* values marked with an asterisk indicate statistical significance (***p < 0.001; ****p < 0.0001). eGFR, estimated glomerular filtration rate; HbA1c, glycated hemoglobin; BMI, body mass index.

When the exploratory cut-off values for discriminating BRS < 0 derived from the SGLT2i group in the internal cohort (HbA1c: 6.6%; eGFR: 65 mL/min/1.73 m^2^) were applied to the external cohort, the prevalence of BRS < 0 tended to increase among patients with baseline HbA1c ≤ 6.6% or baseline eGFR ≤ 65 mL/min/1.73 m^2^, although these differences did not reach statistical significance in Fisher’s exact test (**Figures 5G, H**). When these variables were defined as candidate risk factors, the prevalence of BRS < 0 showed a stepwise trend of increase according to the number of candidate risk factors (0, 1, or 2), although this trend did not reach statistical significance (**Figure 5I**). These findings suggest that lower HbA1c and reduced eGFR may be associated with lower BRS early after initiation of SGLT2i therapy.

## 4 Discussion

In the present study, we analyzed changes in body composition across three treatment groups—SGLT2i, GLP-1RA, and GLP-1RA added to SGLT2i—to investigate treatment-associated patterns in the balance between muscle and fat mass changes. Our findings suggest that, in patients with type 2 diabetes mellitus, lower baseline HbA1c levels or reduced eGFR may be associated with relatively unfavorable body recomposition following SGLT2i therapy, as indicated by lower BRS, an exploratory index of muscle-to-fat balance.

Previous studies have reported different results regarding the body composition changes associated with SGLT2i and GLP-1RAs, ranging from predominantly fat mass loss to disproportionate loss of muscle mass (12). The EMPA-Elderly study evaluated the effects of 52-week treatment with the SGLT2i empagliflozin on muscle mass and strength in Japanese elderly adults with T2DM and demonstrated that empagliflozin improved glucose control and reduced body weight without compromising muscle mass or strength (20, 21). In the EMPA-Elderly study, the placebo-adjusted changes with empagliflozin were −0.61 kg in muscle mass and −1.84 kg in fat mass, respectively. Similarly, our study showed mean changes of −0.37 kg in skeletal muscle mass and −1.95 kg in fat mass, indicating that fat loss predominated on average after SGLT2i treatment.

However, when the correlations between ΔBFM and ΔASM in each individual were examined, no correlation was detected in the SGLT2i or GLP-1-on-SGLT2i groups, suggesting substantial interindividual variability, in contrast with the GLP-1RA group. In the external cohort, ΔBFM and ΔSMM showed a negative correlation, suggesting substantial interindividual heterogeneity in early body recomposition after SGLT2i initiation. To facilitate a simple and intuitive assessment of the relative changes in muscle and fat, we introduced the Body Recomposition Score (BRS = ΔASM − ΔBFM). This exploratory and operational index was intended to summarize the relative direction of two body composition changes measured in the same unit. We operationally defined BRS < 0 as indicating a pattern in which skeletal muscle loss exceeded fat mass reduction or fat mass gain exceeded skeletal muscle gain. BRS requires further clinical validation, taking into account the different physiological and prognostic significance of changes of the same magnitude in ASM and BFM. A related limitation in interpreting BRS is that we focused on treatment groups in which changes in body weight and body composition were expected, namely SGLT2i, GLP-1RA, and GLP-1RA add-on to SGLT2i therapy, and therefore did not explore the natural one-year distribution of BRS in patients with T2DM receiving stable treatment. Although BRS would theoretically be 0 when neither ASM nor BFM changes, the natural variability and distribution of BRS over time remain to be clarified in future studies. In the present internal cohort, 15 patients (20.5%) showed BRS < 0, corresponding approximately to the lower fifth of the observed BRS distribution. We therefore used BRS < 0 as a hypothesis-generating indicator of a lower-tail subgroup with a relatively unfavorable muscle-to-fat balance in subsequent analyses. Whether BRS is associated with muscle strength, physical function, sarcopenia criteria, and prognostic endpoints should be examined in future studies.

Notably, BRS showed significant positive correlations with both baseline HbA1c and eGFR in the SGLT2i group, in contrast to the other two groups. Among patients treated with SGLT2i who had both HbA1c ≤ 6.6% and eGFR ≤ 65 mL/min/1.73 m^2^, BRS < 0 was observed in 80% in the internal cohort and 62.5% in the external cohort, although stratification using the cut-off values derived from the internal cohort was not statistically significant in the external cohort. The continuous associations of BRS with baseline HbA1c and eGFR observed at 12 months in the SGLT2i group of the internal cohort were directionally reproduced at 4 weeks in the external cohort, suggesting that lower BRS may already be detectable early after SGLT2i initiation in patients with low baseline HbA1c or reduced eGFR. These findings also highlight the potential utility of BRS as a simple exploratory indicator for assessing the relative balance between muscle and fat mass changes during therapy. Regarding the positive ΔASM observed in the GLP-1-on-SGLT2i group with low eGFR in the internal cohort, possible explanations may include the modifying effects of GLP-1RA add-on therapy on insulin secretion and glucagon suppression. However, this finding was not accompanied by a significant difference in BRS between eGFR strata and requires further validation.

Although age was not significantly associated with BRS in Pearson correlation analysis in the SGLT2i group of the internal cohort, Spearman’s rank correlation analysis showed a significant negative correlation between age and BRS. A significant negative correlation between age and BRS was also observed in the external cohort, suggesting that older age may be associated with lower BRS after SGLT2i initiation. Prospective studies are warranted to confirm whether age modifies body composition changes after SGLT2i therapy, particularly in patients with low HbA1c or reduced renal function.

From the perspective of inter-organ metabolic regulation, our findings suggest that SGLT2i and GLP-1 receptor agonists may differentially shift the balance between adipose tissue and skeletal muscle, particularly in patients with lower baseline HbA1c or reduced eGFR. Several mechanisms have been proposed for the body composition changes induced by SGLT2i and GLP-1 receptor agonists (10). GLP-1RAs enhance glucose-dependent insulin secretion from pancreatic β-cells and suppress glucagon secretion from α-cells. In contrast, SGLT2i lower glycemic levels in an insulin-independent manner and are known to increase serum glucagon levels, which may limit glucose and amino acid utilization in skeletal muscle and promote hepatic gluconeogenesis and glycogenolysis (1) (11) (22). The observed association between lower baseline HbA1c and lower BRS in patients treated with SGLT2i may be partly explained by a shift in the insulin-to-glucagon balance; in patients with lower baseline HbA1c, sustained SGLT2i-induced glycosuria may be associated with reduced insulin activity and increased glucagon signaling.

Reduced eGFR may further amplify the muscle-to-fat imbalance through chronic kidney disease (CKD)-related catabolic mechanisms. CKD is known to promote protein catabolism and muscle wasting through multiple mechanisms, including defective insulin signaling, metabolic acidosis, enhanced glucocorticoid activity, inflammation, and increased angiotensin II levels (23). These mechanisms may increase amino acid mobilization from skeletal muscle and alter systemic amino acid flux among muscle, kidney, and liver. A previous review proposed that SGLT2i may exploit muscle energy and nitrogen reservoirs for glucose and urea production (24). This framework may link SGLT2i-associated changes in hydration and renal water conservation with amino acid mobilization and systemic energy metabolism. Recently, we demonstrated in a mouse model of type 2 diabetes that SGLT2i treatment restored renal branched-chain amino acid (BCAA) catabolism and improved histopathology of diabetic nephropathy compared with the control treated with insulin exhibiting equivalent glycemic levels (25). Further studies are needed to elucidate how SGLT2i influence systemic amino acid flux across multiple organs, including muscle, kidney, and liver.

Overall, this mechanistic interpretation remains hypothetical and requires further investigation, including comparisons with other glucose-lowering drug classes, such as metformin, DPP-4 inhibitors, thiazolidinediones, insulin, and imeglimin. These agents may also modify the relationship between baseline glycemic status and muscle-to-fat balance during treatment by influencing insulin secretion, insulin sensitivity, mitochondrial metabolism, glucagon activity, amino acid utilization, and protein catabolism.

Several limitations of this study should be acknowledged. First, baseline HbA1c levels differed among treatment groups in the internal cohort. Although BRS distributions were descriptively shown by treatment group, the present retrospective study was not designed or powered to make direct comparative inferences about the effects of each treatment regimen on body recomposition. Baseline glycemic status may therefore have influenced the observed differences in body composition changes among the groups. Prospective randomized controlled trials are required to determine the causal effects of each treatment on body recomposition.

Another limitation is the potential influence of hydration status and fluid distribution on bioelectrical impedance analysis (BIA)-derived body composition estimates. This issue is particularly relevant for SGLT2i therapy because SGLT2 inhibitors can induce osmotic diuresis and alter body fluid balance. In the internal cohort, we found no significant within-group changes in water-related parameters, including intracellular water, extracellular water, and the extracellular water/total body water ratio (**Supplementary Table 1**), suggesting that large fluid shifts were unlikely to fully account for the observed changes in ASM, BFM, or BRS. Nevertheless, these water-related parameters were also derived from BIA, and subtle changes in hydration status or fluid distribution may still have influenced the estimated values of skeletal muscle mass and body fat mass. Therefore, the possibility that fluid shifts partly affected the observed body composition changes cannot be completely excluded. This possibility is particularly relevant to the external cohort, in which body composition was assessed only 4 weeks after SGLT2i initiation. Potential contributors to the observed negative correlation between ΔBFM and ΔSMM in this cohort may include early fluid shifts, as well as interindividual heterogeneity in insulin-to-glucagon balance, gluconeogenesis, amino acid utilization, and catabolic responses.

Finally, although BRS also showed significant positive correlations with baseline HbA1c and eGFR in the external cohort, stratification using the cut-off values derived from the internal cohort was not significantly associated with the prevalence of BRS < 0. Differences in study design, follow-up duration, and body composition assessment methods, including the use of total skeletal muscle mass rather than appendicular skeletal muscle mass in the external cohort, may account for these discrepancies. In addition, because this was a retrospective observational study based on routinely collected clinical data, detailed information on diabetes duration, formally diagnosed comorbidities such as heart failure or liver disease, nutritional intake, physical activity, and concomitant non-diabetic medications was not systematically available. These unmeasured factors, together with insulin action, gluconeogenesis, and hydration status, may have influenced BRS and contributed to residual confounding. The present exploratory findings require further validation in prospective cohorts and the establishment of clinically relevant cut-off values.

In conclusion, our findings suggest that lower baseline HbA1c levels or reduced eGFR may be associated with relatively unfavorable body recomposition after SGLT2i therapy, as assessed by BRS < 0. Although further validation is needed to determine the clinical significance of BRS, these findings support the importance of individualized pharmacotherapy for type 2 diabetes with consideration of muscle-to-fat balance.

## Data Availability

The datasets generated and/or analyzed during the current study are available from the corresponding author upon reasonable request.
