# Supplementary material for: Low baseline HbA1c and reduced eGFR are associated with relatively unfavorable body recomposition after SGLT2 inhibitor therapy in type 2 diabetes
Source: Front Endocrinol (Lausanne). 2026 Aug 27;17:1924744. doi: 10.3389/fendo.2026.1924744 (PMC13557946; doi:10.3389/fendo.2026.1924744)
Supplement: Supplementary file 1 [file Supplementaryfile1.docx]

Supplementary Material

# 1 Supplementary Figure

**Supplementary Figure 1. Distribution of Body Recomposition Score by treatment group and sex.**

Individual data points show the Body Recomposition Score (BRS) in each subgroup. The dotted horizontal line indicates BRS = 0. In panel A, BRS values are shown according to treatment group: SGLT2i (n = 36), GLP-1RA (n = 17), and GLP-1-on-SGLT2i (n = 20). Because normality was not confirmed in all treatment groups, horizontal lines and error bars indicate the median and interquartile range, and between-group differences were assessed using the Kruskal–Wallis test. In panel B, BRS values are shown according to sex: male (n = 38) and female (n = 35). Because normality was confirmed by the Shapiro–Wilk test, horizontal lines and error bars indicate the mean and standard deviation, and the comparison between male and female patients was performed using an unpaired t-test. P values are shown in each panel.

# 2 Supplementary Tables

**Table S1. Clinical and laboratory parameters of study participants at baseline and 12 months (N = 73)**

|  | Within-group analyses | | | | | | | | | | | | | | | | | | | Between-group analyses | | | | | | |  |
| --- | --- | --- | --- | --- | --- | --- | --- | --- | --- | --- | --- | --- | --- | --- | --- | --- | --- | --- | --- | --- | --- | --- | --- | --- | --- | --- | --- |
|  | SGLT2i (n = 36) | | | | | | GLP-1RA (n = 17) | | | | | | GLP-1-on-SGLT2i (n = 20) | | | | | |  | | | *p* | | *p* | |  |  |
|  | baseline | | 12M | | *p* | | baseline | | 12M | | *p* | | baseline | | 12M | | *p* | |  | | | (baseline) | | | (12M) | |  |
| HbA1c (%) | | 7.6±1.2 | | 7.1±0.7 | | 0.0043** | | 9.0±2.0 | | 8.1±1.7 | | 0.0061** | | 8.1±1.0 | | 7.5±1.3 | | 0.0035** | | |  | | 0.0035** | | 0.0198* | | |
| HbA1c  [mmol/mol] | | 59 ± 13 | | 54 ± 8 | |  | | 75 ± 22 | | 65 ± 19 | |  | | 65 ± 11 | | 58 ± 14 | |  | | |  | |  | |  | | |
| BUN (mg/dL) | | 15.2±6.4 | | 17.1±8.5 | | 0.0479* | | 15.3±4.8 | | 15.7±5.3 | | 0.6779 | | 17.0±4.5 | | 18.1±7.5 | | 0.8442 | | |  | | 0.5098 | | 0.6308 | | |
| Cr (mg/dL) | | 0.84±0.24 | | 0.87±0.32 | | 0.9764 | | 0.83±0.37 | | 0.86±0.39 | | 0.3344 | | 0.81±0.24 | | 0.83±0.33 | | 0.9542 | | |  | | 0.9445 | | 0.9448 | | |
| eGFR(mL/min/1.73 m^2^) | | 68.1±18.3 | | 67.7±20.1 | | 0.6117 | | 71.8±27.0 | | 68.1±24.7 | | 0.0551 | | 75.1±25.7 | | 74.0±23.7 | | 0.8270 | | |  | | 0.5330 | | 0.5972 | | |
| AST (IU/L) | | 30.4±23.4 | | 23±7.9 | | 0.0083** | | 23.8±9.3 | | 25.4±16.1 | | 0.9641 | | 30.0±12.7 | | 21.7±4.5 | | 0.0022** | | |  | | 0.4530 | | 0.5312 | | |
| ALT (IU/L) | | 34.2±27.5 | | 25.9±16.7 | | 0.0038** | | 26.8±12.6 | | 26.7±18.6 | | 1.0000 | | 39.7±22.2 | | 26.1±13.4 | | 0.0001*** | | |  | | 0.2747 | | 0.9864 | | |
| γ-GTP (IU/L) | | 58.5±53.4 | | 48.7±60.1 | | 0.0055** | | 36.4±24.7 | | 34.9±28.4 | | 0.7339 | | 60.1±71.2 | | 36.1±35.4 | | 0.0010** | | |  | | 0.3167 | | 0.5351 | | |
| UA (mg/dL) | | 8.7±5.9 | | 9.3±7.7 | | 0.7031 | | 5.5±1.3 | | 5.5±1.3 | | 0.965 | | 5.4±1.8 | | 5.2±1.5 | | 0.3034 | | |  | | 0.0249* | | 0.0397* | | |
| TG (mg/dL) | | 151±101 | | 108±39 | | 0.0429* | | 199±92 | | 181±65 | | 0.8552 | | 150±67 | | 139±75 | | 0.1444 | | |  | | 0.3182 | | 0.3707 | | |
| LDL-C (mg/dL) | | 108±39 | | 112±30 | | 0.6517 | | 115±32 | | 110±25 | | 0.4968 | | 118±22 | | 109±23 | | 0.0583 | | |  | | 0.5329 | | 0.9323 | | |
| HDL-C (mg/dL) | | 53±19 | | 53±12 | | 0.1294 | | 48±15 | | 50±15 | | 0.5020 | | 54±10 | | 53±10 | | 0.0521 | | |  | | 0.5269 | | 0.8157 | | |
|  | |  | |  | |  | |  | |  | |  | |  | |  | |  | | |  | |  | |  | | |
| Body weight (kg) | | 69.1±14.4 | | 66.8±14.7 | | 0.0002*** | | 76.7±17.5 | | 74.9±17.3 | | 0.0431* | | 77.9±12.9 | | 75.6±12.9 | | 0.0033** | | |  | | 0.0570 | | 0.0592 | | |
| BMI (kg/m^2^) | | 27.0±4.7 | | 26.1±4.7 | | 0.0001*** | | 29.7±5.9 | | 29.0±5.9 | | 0.0505 | | 29.1±3.7 | | 28.2±4.4 | | 0.0064** | | |  | | 0.1134 | | 0.1021 | | |
| ASM (kg) | | 18.5±4.1 | | 18.2±4.3 | | 0.0686 | | 19.6±5.1 | | 19.3±4.9 | | 0.4038 | | 21.0±4.4 | | 20.7±4.2 | | 0.3683 | | |  | | 0.2221 | | 0.1950 | | |
| Body fat mass (kg) | | 23.8±9.5 | | 21.9±9.9 | | 0.0003*** | | 30.2 ±11.9 | | 28.4±11.5 | | 0.0128* | | 28.2±7.5 | | 26.3±8.9 | | 0.0086** | | |  | | 0.0573 | | 0.0671 | | |
| SMI (kg/m²) | | 7.2±1.1 | | 7.1±1.2 | | 0.0621 | | 7.5±1.4 | | 7.4±1.3 | | 0.4038 | | 7.8±1.2 | | 7.7±1.1 | | 0.3884 | | |  | | 0.2377 | | 0.1680 | | |
| ICW (L) | | 20.6±4.0 | | 20.3±4.1 | | 0.0715 | | 20.6±4.6 | | 20.5±4.2 | | 0.5906 | | 22.6±4.28 | | 21.8±4.12 | | 0.2089 | | |  | | 0.9165 | | 0.9578 | | |
| ECW (L) | | 12.9±2.2 | | 12.8±2.3 | | 0.4504 | | 13.4±3.1 | | 13.4±2.9 | | 0.9080 | | 14.2±2.6 | | 14.1±2.43 | | 0.7525 | | |  | | 0.2028 | | 0.1718 | | |
| ECW/TBW | | 0.386±0.01 | | 0.388±0.01 | | 0.0785 | | 0.393±0.01 | | 0.395±0.01 | | 0.1950 | | 0.418±0.14 | | 0.393±0.03 | | 0.4698 | | |  | | 0.2995 | | 0.3314 | | |

Table S1. Clinical and laboratory parameters of study participants at baseline and 12 months (12M) in the SGLT2i (n = 36), GLP-1RA (n = 17), and GLP-1RA add-on to SGLT2i (n = 20) groups. Data are presented as mean ± standard deviation. Within-group comparisons were performed using Wilcoxon’s signed-rank test, and between-group comparisons were conducted using one-way ANOVA. Body composition was measured using bioelectrical impedance analysis (BIA) with InBody 770. Statistical significance was defined as p < 0.05. *p < 0.05, **p < 0.01, ***p < 0.001. Abbreviations: HbA1c, hemoglobin A1c; BUN, blood urea nitrogen; Cr, creatinine; eGFR, estimated glomerular filtration rate; AST, aspartate aminotransferase; ALT, alanine aminotransferase; γ-GTP, gamma-glutamyl transpeptidase; UA, uric acid; TG, triglycerides; LDL-C, low-density lipoprotein cholesterol; HDL-C, high-density lipoprotein cholesterol; BMI, body mass index; ASM, appendicular skeletal muscle mass; SMI, skeletal muscle mass index; ICW, intracellular water; ECW, extracellular water; TBW, total body water.

**Table S2. Associations between treatment-associated changes in fat and muscle and baseline characteristics**

|  | SGLT2i (n = 36) | | | | GLP-1RA (n = 17) | | | | GLP-1-on-SGLT2i (n = 20) | | | |
| --- | --- | --- | --- | --- | --- | --- | --- | --- | --- | --- | --- | --- |
|  | ΔASM (kg) | | ΔBFM (kg) | | ΔASM (kg) | | ΔBFM (kg) | | ΔASM (kg) | | ΔBFM (kg) | |
| Baseline values | PCC | *p* | PCC | *p* | PCC | *p* | PCC | *p* | PCC | *p* | PCC | *p* |
| Age (years) | -0.28 | 0.10 | 0.10 | 0.55 | 0.09 | 0.72 | 0.18 | 0.49 | 0.13 | 0.59 | 0.11 | 0.65 |
| Body weight (kg) | -0.05 | 0.78 | 0.07 | 0.68 | -0.13 | 0.61 | -0.37 | 0.15 | -0.17 | 0.48 | 0.41 | 0.07 |
| Body fat mass (kg) | -0.16 | 0.33 | -0.01 | 0.92 | 0.09 | 0.72 | -0.27 | 0.29 | 0.15 | 0.52 | 0.30 | 0.20 |
| ASM (kg) | 0.05 | 0.75 | 0.14 | 0.41 | -0.28 | 0.28 | -0.27 | 0.30 | -0.32 | 0.17 | 0.35 | 0.13 |
| Hemoglobin A1c (%) | 0.02 | 0.89 | -0.36 | 0.03* | -0.07 | 0.78 | -0.13 | 0.62 | 0.05 | 0.82 | -0.31 | 0.19 |
| eGFR (mL/min/1.73 m^2^) | 0.08 | 0.63 | -0.31 | 0.06 | -0.05 | 0.84 | -0.08 | 0.75 | -0.15 | 0.53 | -0.18 | 0.44 |

Table S2. Correlations between treatment-associated changes in appendicular skeletal muscle mass (ΔASM) and fat mass (ΔBFM) and baseline clinical parameters were analyzed using Pearson’s correlation coefficient in the SGLT2i, GLP-1RA, and GLP-1RA add-on to SGLT2i groups. A two-tailed p value < 0.05 was considered statistically significant. *p < 0.05. Abbreviations: SGLT2i, sodium-glucose cotransporter 2 inhibitor; GLP-1RA, glucagon-like peptide-1 receptor agonist; ΔASM, change in appendicular skeletal muscle mass; ΔBFM, change in body fat mass; PCC, Pearson correlation coefficient; ASM, appendicular skeletal muscle mass.

**Table S3. Correlations between Body Recomposition Score (BRS) and baseline characteristics**

|  | SGLT2i (n = 36) | | GLP-1RA (n = 17) | | GLP-1-on-SGLT2i (n = 20) | |
| --- | --- | --- | --- | --- | --- | --- |
| Baseline values | PCC | *p* | PCC | *p* | PCC | *p* |
| Age (years) | -0.21 | 0.22 | -0.18 | 0.49 | -0.07 | 0.78 |
| Body weight (kg) | -0.09 | 0.60 | 0.40 | 0.12 | -0.44 | 0.05 |
| Body fat mass (kg) | -0.05 | 0.79 | 0.39 | 0.11 | -0.24 | 0.30 |
| ASM (kg) | -0.12 | 0.49 | 0.20 | 0.45 | -0.42 | 0.06 |
| Hemoglobin A1c (%) | 0.36 | 0.03* | 0.13 | 0.63 | 0.31 | 0.19 |
| eGFR (mL/min/1.73 m^2^) | 0.34 | 0.04* | 0.10 | 0.76 | 0.13 | 0.57 |

Table S3. Correlations between Body Recomposition Score (BRS = ΔASM – ΔBFM) and baseline clinical parameters were analyzed using Pearson’s correlation coefficient in the SGLT2i, GLP-1RA, and GLP-1RA add-on to SGLT2i groups. A two-tailed p value < 0.05 was considered statistically significant. *p < 0.05.

Abbreviations: PCC, Pearson’s correlation coefficient; ASM, appendicular skeletal muscle mass; BFM, body fat mass; SGLT2i, sodium-glucose cotransporter 2 inhibitor; GLP-1RA, glucagon-like peptide-1 receptor agonist.

**Table S4. Spearman’s rank correlations between Body Recomposition Score (BRS) and baseline characteristics**

|  | SGLT2i (n = 36) | | GLP-1RA (n = 17) | | GLP-1-on-SGLT2i (n = 20) | |
| --- | --- | --- | --- | --- | --- | --- |
| Baseline values | ρ | p | ρ | p | ρ | p |
| Age (years) | -0.39 | 0.018* | 0.05 | 0.851 | 0.13 | 0.594 |
| Body weight (kg) | 0.08 | 0.642 | -0.42 | 0.068 | 0.35 | 0.165 |
| Body fat mass (kg) | 0.10 | 0.575 | -0.23 | 0.339 | 0.28 | 0.277 |
| ASM (kg) | -0.01 | 0.961 | -0.39 | 0.085 | 0.09 | 0.743 |
| Hemoglobin A1c (%) | 0.37 | 0.027* | 0.05 | 0.848 | 0.40 | 0.077 |
| eGFR (mL/min/1.73 m^2^) | 0.42 | 0.011* | -0.10 | 0.694 | -0.10 | 0.682 |

Table S4. Values are Spearman’s rank correlation coefficients (ρ) and corresponding p values. *p < 0.05. ASM, appendicular skeletal muscle mass; BFM, body fat mass; BRS, Body Recomposition Score; eGFR, estimated glomerular filtration rate; SGLT2i, sodium-glucose cotransporter 2 inhibitor; GLP-1RA, glucagon-like peptide-1 receptor agonist.

**Table S5. AUC values and exploratory cut-off points for discriminating BRS < 0**

|  | Baseline parameters | Optimal cut-off values | *p*-value | AUC | Sensitivity | Specificity |
| --- | --- | --- | --- | --- | --- | --- |
|  | HbA1c (% [mmol/mol]) | 6.6 [49] | 0.002** | 0.94 | 0.97 | 0.90 |
| SGLT2 inhibitor | eGFR (mL/min/1.73 m^2^) | 65 | 0.049* | 0.78 | 1.00 | 0.55 |
|  | Age (years) | 65 | 0.599 | 0.57 | 0.80 | 0.52 |
| GLP-1RA | HbA1c (% [mmol/mol]) | 10.3 [89] | 0.712 | 0.56 | 1.00 | 0.33 |
|  | eGFR (mL/min/1.73 m^2^) | 61 | 0.429 | 0.62 | 1.00 | 0.42 |
|  | Age (years) | 66 | 0.598 | 0.58 | 0.80 | 0.58 |
| GLP-1-on-SGLT2i | HbA1c (% [mmol/mol]) | 7.9 [62] | 0.018* | 0.86 | 1.00 | 0.67 |
|  | eGFR (mL/min/1.73 m^2^) | 28 | 0.760 | 0.54 | 0.20 | 1.00 |
|  | Age (years) | 48 | 0.760 | 0.55 | 0.40 | 0.87 |

Table S5. Area under the curve (AUC) and exploratory cut-off values were determined by receiver operating characteristic (ROC) curve analysis to discriminate relatively unfavorable body recomposition, defined as a Body Recomposition Score (BRS; ΔASM − ΔBFM) < 0, in each treatment group. *p < 0.05, **p < 0.01 compared with an AUC of 0.5.

Abbreviations: BRS, Body Recomposition Score; ASM, appendicular skeletal muscle mass; BFM, body fat mass; eGFR, estimated glomerular filtration rate; HbA1c, hemoglobin A1c; SGLT2i, sodium-glucose cotransporter 2 inhibitor; GLP-1RA, glucagon-like peptide-1 receptor agonist.

**Table S6. Comparison of BRS and other body composition–related parameters between patients stratified by baseline HbA1c (≤ 6.6% vs > 6.6%) or baseline eGFR (≤ 65 vs > 65 mL/min/1.73 m²) in each treatment group**

|  | HbA1c ≤ 6.6 | HbA1c > 6.6 | *p* | eGFR ≤ 65 | eGFR > 65 | *p* |
| --- | --- | --- | --- | --- | --- | --- |
| **SGLT2 inhibitor** | n = 5 | n = 31 |  | n = 19 | n = 17 |  |
| BRS | -2.23±2.97 | 2.13±2.07 | **0.0008*** | 0.56±3.02 | 2.60±1.67 | **0.0177*** |
| ΔASM | -0.41±0.92 | -0.33±1.05 | 0.9248 | -0.43±1.17 | -0.24±0.86 | 0.5783 |
| ΔBFM | 1.82±2.62 | -2.46±2.14 | **0.0018*** | -1.0±3.04 | -2.84±1.71 | **0.0474*** |
| ASM baseline | 20.06±3.16 | 18.33±4.19 | 0.4642 | 19.68±3.49 | 17.34±4.41 | 0.0884 |
| ASM 12M | 19.65±3.58 | 18.0±4.35 | 0.4783 | 19.25±3.58 | 17.10±4.74 | 0.1829 |
| BFM baseline | 21.10±5.22 | 24.20±10.06 | 0.5571 | 24.89±8.47 | 22.52±10.73 | 0.3924 |
| BFM 12M | 22.92±5.91 | 21.73±10.42 | 0.5692 | 23.89±9.16 | 19.67±10.40 | 0.1195 |
| BMI baseline | 26.52±2.35 | 27.12±4.94 | 0.9647 | 27.84±4.38 | 26.13±4.90 | 0.2575 |
| BMI 12M | 26.87±3.14 | 25.98±4.92 | 0.4759 | 27.20±4.47 | 24.88±4.74 | 0.0933 |
| **GLP-1RA** | n = 1 | n = 16 |  | n = 7 | n = 10 |  |
| BRS | 4.0 | 1.34±1.91 | NA | 1.72±1.63 | 1.34±2.23 | 0.4173 |
| ΔASM | -2.0 | -0.16±0.93 | NA | -0.21±1.13 | -0.31±0.98 | 0.8868 |
| ΔBFM | -6.0 | -1.51±2.30 | NA | -1.94±1.97 | -1.66±2.89 | 0.4592 |
| ASM baseline | 32.6 | 18.77±3.99 | NA | 19.40±5.05 | 19.71±5.43 | >0.9999 |
| ASM 12M | 30.6 | 18.61±4.12 | NA | 19.19±5.30 | 19.40±4.96 | 0.8868 |
| BFM baseline | 46.0 | 29.18±11.55 | NA | 32.23±12.89 | 28.72±11.63 | 0.8868 |
| BFM 12M | 40.0 | 27.66±11.44 | NA | 30.29±13.05 | 27.06±10.76 | 0.7582 |
| BMI baseline | 39.8 | 29.02±5.41 | NA | 30.25±6.50 | 29.23±5.69 | >0.9999 |
| BMI 12M | 38.4 | 28.35±5.54 | NA | 29.33±6.46 | 28.67±5.80 | 0.9623 |
| **GLP-1-on-SGLT2i** | n = 1 | n = 19 |  | n = 5 | n = 15 |  |
| BRS | -2.5 | 1.94±3.11 | NA | 2.69±3.41 | 1.40±3.16 | 0.4342 |
| ΔASM | 0.0 | -0.23±0.89 | NA | 0.51±0.67 | -0.46±0.80 | **0.0254*** |
| ΔBFM | 2.5 | -2.17±2.92 | NA | -2.19±3.72 | -1.86±2.91 | 0.6567 |
| ASM baseline | 24.6 | 20.77±4.40 | NA | 18.98±4.42 | 21.63±4.30 | 0.2661 |
| ASM 12M | 24.6 | 20.54±4.18 | NA | 19.50±4.18 | 21.16±4.23 | 0.5531 |
| BFM baseline | 34.3 | 27.89±7.57 | NA | 30.78±5.27 | 27.36±8.09 | 0.4973 |
| BFM 12M | 36.8 | 25.71±8.78 | NA | 28.60±8.04 | 25.49±9.29 | 0.8001 |
| BMI baseline | 33.5 | 28.86±3.68 | NA | 29.57±4.14 | 28.94±3.73 | >0.9999 |
| BMI 12M | 34.4 | 27.90±4.29 | NA | 29.24±5.48 | 27.89±4.18 | **0.0328*** |

Table S6. BRS and other body composition–related parameters were compared between patients stratified by baseline HbA1c (≤ 6.6% vs > 6.6%) and, separately, by baseline eGFR (≤ 65 vs > 65 mL/min/1.73 m²), using the Mann–Whitney U test. NA indicates that statistical testing was not applicable due to small sample size. A two-tailed p value < 0.05 was considered statistically significant. *p < 0.05.

Abbreviations: ASM, appendicular skeletal muscle mass; BFM, body fat mass; BMI, body mass index; BRS, Body Recomposition Score; eGFR, estimated glomerular filtration rate; GLP-1RA, glucagon-like peptide-1 receptor agonist; HbA1c, hemoglobin A1c; NA, not applicable; SGLT2i, sodium-glucose cotransporter 2 inhibitor; 12M, 12 months after treatment.
